# Estimation of Entropy Generation in a SCR-DeNOx System with AdBlue Spray Dynamic Using Large Eddy Simulation

**DOI:** 10.3390/e25030475

**Published:** 2023-03-09

**Authors:** Kaushal Nishad, Senda Agrebi

**Affiliations:** 1 Institute of Reactive Flows and Diagnostics, Department of Mechanical Engineering, Technical University of Darmstadt, Otto-Berndt-Str. 3, 64287 Darmstadt, Germany; 2 Institute of Energy and Power Plant Technology, Department of Mechanical Engineering, Technical University of Darmstadt, Otto-Berndt-Str. 3, 64287 Darmstadt, Germany

**Keywords:** selective catalytic converter, large eddy simulation, entropy production, AdBlue injection, spray dynamics, wall-film

## Abstract

In this work, the entropy generation analysis is extended to the multi-phase fluid flow within a Large Eddy Simulation (LES) framework. The selected study case consists of a generic selective catalytic reduction (SCR) configuration in which the water/AdBlue is injected into a cross-flow of the internal combustion (IC) engine exhaust gas. The adopted numerical modules are first assessed by comparing with experimental data for film thickness in the case of AdBlue injection and then with H_2_O mass fraction and temperature for water injection case. Subsequently, the impact of heat transfer, fluid flow, phase change, mixing and chemical reaction due to AdBlue injection on the entropy generation is assessed. Hence, the individual contributions of viscous and heat dissipation together with the species mixing, chemical reaction during the thermal decomposition of urea into NH_3_ and dispersed phase are especially evaluated and analysed. In comparison to the shares of the viscous and mixing processes, the entropy generation is predominated by the heat, chemical and dispersed phase contributions. The influence of the operating parameters such as exhaust gas temperature, flow rate and AdBlue injection on entropy generation is discussed in details. Using a suitable measures, the irreversibility map and some necessary inferences are also provided.

## 1. Introduction

Currently, intense focus is being put on the technologies to make the modern automobiles more environmentally friendly—especially in term of emission of the particulate matter and flue gas such as greenhouse gas (CO_2_), NO_X_, SO_X_ etc. In particular, to cut down the greenhouse gas, the use of the so-called carbon neutral fuels are being promoted. The existing power-trains with conventional fuels are expected to be replaced by bio-fuels or low carbon fuels such as natural gas, liquid petroleum gas, methanol, hydrogen, synthetic fuels, E-fuels, etc. [1,2,3,4]. Additionally, the battery operated electric vehicles (BEVs) are considered to be promising and cleaner transport alternative. However, in the foreseeable future it is not expected that the BEVs provide any complete replacement of the traditional power-trains. In fact, depending on the source of renewable energy and fuel availability locally, various power-trains will coexist to drive the respective vehicles [5], with additional focus on existing power-trains operating with alternative carbon neutral fuels with enhanced in-cylinder technology. However, even the most advanced and efficient in-cylinder technologies alone can not guarantee the compliance to the prescribed emission norms for the power-train in use. In this regard, the exhaust-gas-after-treatment system (EGAS) provides the complementary solution to this issue in which the harmful exhaust species are either oxidised or reduced into harmless substances downstream/outside the (IC) engine. The stricter emission norms for the present and future automobiles sought for even more advanced and efficient EGAS. In particular, the higher concentration of NO_X_ in the exhaust gas of a compression ignition (CI) engine considerably offsets its advantageous characteristics such as relatively more powerful engine, better fuel efficiency and less CO_2_ emission as compared to automobiles operated by gasoline-like fuels. In this regard, selective catalytic reduction (SCR) is considered a vital and proven EGAS to reduce the engine NO_X_ and meet the targeted emission norm of a particular automobile.

Focusing on computational fluid dynamics (CFD) based numerical analysis and design optimization of EGAS, the Reynolds-averaged Navier–Stokes (RANS)-based methods were the most adopted approach as it can provide a macroscopic understanding of the relevant processes with relatively less computational expenses and time [6,7,8]. However, the process inside a SCR system features a highly unsteadiness starting from the hot turbulent exhaust gas from the engine manifold, AdBlue injection, AdBlue film development, deposit formation and ammonia conversion to the NO_X_ reduction. Additionally the history effect due to AdBlue film build up and formation of solid-deposit in SCR duct demands a numerical simulation of many consecutive engine cycles for longer physical time. These are individually very complex processes and their mutual interaction makes the development of numerical models for an SCR system even more challenging task. The lack of comprehensive reference or experimental data to validate the adopted numerical methods for a given SCR system presents additional challenges to the modelling communities. Therefore, Payri et al. [8] firstly, validated the adopted atomization model by using the in-house data. Subsequently, the available data for NH_3_ conversion in another realistic SCR duct [9] was utilized to evaluate the used reacting kinetics of urea decomposition and NH_3_ conversion for the RANS simulation. Similarly, based on the Large Eddy Simulation (LES) modelling approach, Nishad et al. [10,11,12] investigated the influence of cross-flow rate and temperature on the spray dynamics in a realistic SCR system, whereas [13] adopted the so called hybrid LES-RANS approach in order to resolve adequately the AdBlue injection in opposite to gas flow direction.

As stated before, a SCR system features complex and coupled multi-phase reacting flow phenomena, making both the numerical analysis and design/process optimization a difficult task. Nevertheless, the CFD based numerical analysis can still provide important guidelines in the selection of optimal AdBlue dosing strategy, mixer plate and system layout of exhaust-after-treatment under varied operating conditions. In this regard, the entropy generation based irreversibility analysis which is known to be a useful tool (see [14,15,16,17] and therein quoted papers), can be implicated especially in identifying the key processes to control the efficient operation of the SCR system. In term of thermodynamic efficiency, the exergy loss in a system is proportional to the total entropy production [18,19]. Thus, the design or process optimization of a real industrial systems can be accomplished based on entropy generation minimization (EGM) approach. Moreover, the suitability of entropy generation-based approach in providing a deeper insight about the coupled thermo-fluid flow processes is also reported in our previous works [20,21] using LES-based detailed description. Additionally, such a method has been successfully utilized to investigate various technical configurations and physical processes, as reported in [17,19,21,22,23,24,25,26,27,28,29,30,31,32,33]. More recently, the entropy generation based analysis of monolithic catalytic converter using LES was carried out by Li et al. [34] to understand especially the turbulent flow transition and to characterize the single phase flow inside the SCR monolith, namely the impinging flow with stagnation, re-circulation, flow separation and laminarization. A recent review can be found in [35] related to the entropy generation analysis in thermo-fluid systems which involve chemical reactions or combustion. However, the aspects under consideration of multi-phase flow phenomena have not yet been reported.

Therefore, the objective of this paper is to extend the entropy generation analysis to multi-phase fluid flow within a Large Eddy Simulation framework. The selected study case consists of a generic selective catalytic reduction configuration in which AdBlue is injected into a cross-flow of IC engine exhaust gas. In particular, the impact of heat transfer, fluid flow, phase change, mixing and chemical reaction due to AdBlue injection on the entropy generation is assessed. Consequently, the individual contributions of viscous and heat dissipation together with the species mixing, chemical reaction during the thermal decomposition of urea into NH_3_ and dispersed phase are quantified and analysed. The influence of the operating parameters such as exhaust gas temperature, flow rate and AdBlue injection on entropy generation is discussed in details. Using suitable measures, the irreversibility map and some necessary inferences are also provided.

For this purpose, this paper is organized as follows. First, the relevant numerical methods are briefly outlined in Section 2. The generic SCR configuration, the operating parameters and necessary numerical setups are then described in Section 3. After an appropriate validation of the reacting flow properties, detailed and comprehensive analysis and discussion of the obtained numerical results are provided in Section 4. Finally, the key outcomes from this investigation are highlighted in the last Section 5.

## 2. Numerical Methodology

In the present work, the numerical simulation is carried out using an open source numerical code OpenFOAM-v1612+ [36]. The modelling of hot carrier gas flow in a generic SCR configuration with AdBlue injection is conducted based on an Eulerian–Lagrangian approach within large eddy simulation (LES) framework. The Eulerian framework is dedicated to the carrier phase in which the turbulent flow is modelled using the one-equation sub-grid scale (SGS) model as proposed in [37] to close the SGS stress tensor in the filtered equation of momentum, while a simple gradient approach to close the SGS scalar flux in the filtered governing equation of the scalar fields is applied. The Lagrange particle tracking method is used to solve the droplet motions and related spray dynamics. The species evolution during the droplet evaporation is traced by well-proven multi-component droplet evaporation model [10,11]. The thermal decomposition of the resulting gaseous urea is described by the two-step chemical reaction mechanism as reported in [38]. The accurate representation of the complex turbulence–chemistry–particle interaction is realized by an Eulerian–Stochastic Field (ESF) method as reported [39,40,41]. Further, the spray–wall interaction and AdBlue wall film formation are realized by the combination of thin-film and necessary droplet interaction by the so called the interaction regime map [42,43] by further accounting the peculiar behaviour of AdBlue droplet–wall interactions as reported in [44]. Since, the processes inside a catalytic converter is highly complex in nature with interacting phenomena such as hot turbulent exhaust flow, AdBlue injection and spray dynamics, droplet/film evaporation and chemical reactions, the entropy generation based analysis of a generic SCR system is then carried out to identify the contributions of the involved individual process in the system irreversibilities. These numerical models are provided in the following sections with more details.

### 2.1. Lagrangian Droplet Tracking

A brief description of the investigated droplet motion equation and multi-component droplet evaporation model is presented in this section. The equations for droplet position $x_{i}$ and velocity $v_{i}$ are based on a Lagrangian formulation and provided by a set of ordinary differential equations as:

(1) $$\frac{dx_{i}}{dt}=v_{i},\frac{dv_{i}}{dt}=\frac{1}{m_{d}}\sum_{i}F_{i}=\frac{1}{m_{d}}\left[F_{D}+F_{g}\right]$$

(2) $$F_{g}=m_{d}g\left(1-\frac{\rho }{\rho_{d}}\right),F_{D}=\frac{C_{D}}{\tau_{d}}\frac{Re_{d}}{24}\left(u_{i}-v_{i}\right)$$

By considering the fact that the density ratio of droplet liquid and the carrier phase is in the order of ≈$10^{3}$, only the drag $F_{D}$ and gravitational forces $F_{g}$ are considered while the effect of Soret force, Baset force and force acting on particle due to pressure gradient are neglected. The quantity $\tau_{d}=\rho_{p}d_{p}^{2}/\left(18\mu_{g}\right)$ expresses the droplet relaxation time, $Re_{d}=\left|u_{i}-v_{i}\right|d_{p}\rho_{g}/\mu_{g}$ is the droplet Reynold’s Number with $u_{i}$ is the carrier phase velocity vector and $C_{D}$ is the droplet drag coefficient. The latter is defined as;

(3) $$C_{D}=\left\{\begin{array}{cc} \frac{24}{Re_{d}}\left(1+\frac{1}{6}{Re_{d}}^{1/3}\right) & \mathrm{if}\;Re_{d}<1000\\ 0.424 & \mathrm{if}\;Re_{d}\geq 1000 \end{array}\right.$$

Based on an uniform temperature model for the droplet interior, the heat and mass exchanges with the gaseous phase are computed following the model proposed by Miller et al. [45]. In particular, the evaporation rate is described by:

(4) $${\dot{m}}_{d}=\frac{dm_{d}}{dt}=\sum_{i}^{N_{s}}{\dot{m}}_{i}=\sum_{i}^{N_{s}}\left[\pi d_{d}{\left(\bar{\rho }\bar{D}\right)}_{i,g}Sh_{i}ln\left(1+B_{M,i}\right)\right],$$

and the heat balance is given by:

(5) $$\frac{dT_{d}}{dt}=-\frac{1}{m_{d}C_{p,d}}\left(Q+\sum_{i}^{N_{s}}{\dot{m}}_{i}H_{vap,i}\right)=-\frac{\dot{m}C_{p,vap,ref}\left(T_{g}-T_{d}\right)/B_{T}-\sum_{i}^{N_{s}}{\dot{m}}_{i}H_{vap,i}}{m_{d}C_{p,d}}$$

In these equations, ${\dot{m}}_{d}$ represents the total evaporation rate of droplet, ${\dot{m}}_{i}$ is the evaporation rate of individual species *i*, $d_{d}$ is the droplet diameter and $D_{i,g}$ is the binary diffusion of component *i* in the gas. $T_{d}$ represents the droplet temperature, $C_{p,vap,ref}$ and $C_{p,d}$ are specific heat capacities of vapour phase and droplets, $H_{vap}$ is the latent heat and $B_{T}$ the Spalding heat transfer number defined as

(6) $$B_{T}={\left(1+B_{M}\right)}^{\frac{1}{Le}\frac{C_{p,d}}{C_{p,vap,ref}}},$$

where Le, the gas film Lewis number is taken as unity in this study, and $B_{M}=\sum_{i}^{Ns}B_{M,i}$, the Spalding mass transfer, which is in turn expressed for individual species as:

(7) $$B_{M,i}=\frac{Y_{i,s}-Y_{i,\infty }}{1-Y_{i,s}}$$

and

(8) $${Sh}_{i}=2.0+0.6Re^{1/2}Sc_{i}^{1/3}$$

Here, Re and $Sc_{i}$ are the Reynolds and Schmidt number, respectively, defined as:

(9) $$Re=\frac{\rho V_{rel}D}{\mu }\;Sc_{i}=\frac{\mu_{g}}{\rho_{g}D_{i}g},$$

where $Y_{i,s}$ and $Y_{i,\infty }$ are the mass fraction at the droplet surface and far from the droplet surface, respectively.

The evaporation rate can be related to the heat transfer correlation as [46];

(10) $$\dot{m}=2\pi r_{d}\frac{\lambda_{g}}{C_{pg}}Nu\ln\left(1+B_{T}\right)$$

where $\lambda_{g}$ is the heat conductivity of gaseous media, and Nu the dimensionless Nusselt number defined as [46];

(11) $$Nu=2.0+0.6Re^{1/2}Pr^{1/3}\;Pr=\frac{C_{pg}\mu_{g}}{\lambda_{g}},$$

The quantity Pr is the Prandtl number, $C_{pg}$, $\mu_{g}$ and $\lambda_{g}$ are the specific heat, the viscosity and the thermal conductivity of carrier gas, respectively. It should be noted here that in case of static and zero-gravity evaporation with no droplet relative velocity, the value of correlations for both Sh and Nu becomes 2.

The two-way coupling between the carrier phase and the spray droplets (or parcels) are accomplished by the mass, momentum and energy exchanges between the phases. This is realized by the introduction of the respective source terms in mass, species, momentum and energy transport equations within the LES framework due to the presence of spray droplets in the carrier phase control volumes as follows:

(12) $${\bar{S}}_{I,i}=\frac{1}{V}\sum_{j}^{N_{p}}N_{d}{\dot{m}}_{d,j,i}\;{\bar{S}}_{I}=\sum_{i}^{N_{s}}S_{I,i},$$

(13) $${\bar{S}}_{II}=\frac{1}{V}\sum_{j}^{N_{p}}N_{d}\left(-F_{D,j}+m_{d,j}v_{d,j}\right),$$

(14) $${\bar{S}}_{III}=\frac{1}{V}\sum_{j}^{N_{p}}N_{d}\left[-F_{D,j}v_{d,j}+Q+m_{d,j}\frac{1}{2}v_{d,j}v_{d,j}+\sum_{i}^{N_{s}}{\dot{m}}_{i}H_{vap,i}\right],$$

The quantities ${\bar{S}}_{I,i}$, ${\bar{S}}_{I}$, ${\bar{S}}_{II}$, ${\bar{S}}_{III}$ represent the source terms in species, mass, momentum and heat transport equations, respectively. $N_{s}$ the number of species in droplet (multi-component droplet), $N_{d}$ is the number of real droplet in a parcel, $N_{p}$ the total number of parcels present in a given carrier phase control volume.

### 2.2. Thermal Decomposition

With the help of the available heat in the exhaust gas, a solution of 32.5% urea in water (AdBlue) is sprayed into the exhaust duct ahead of the SCR catalyst. Thus, the water is evaporated as:

(15) $${CO\left(NH_{2}\right)}_{2\left(aq\right)}\to {CO\left(NH_{2}\right)}_{2\left(s,l\right)}+H_{2}O_{\left(g\right)}\;.$$

The resulting urea, if in solid state first melts (melting point is at 407 K) and, starts to decompose thermally. According to Koebel et al. [47], the decomposition produces ammonia accompanied by the formation of biuret, triuret and ammonium isocyanate. Above 453 K, cyanuric acid and other compounds of higher molecular weight are produced. In particular, if the urea heating is very fast, the above reactions under loss of ammonia are suppressed and the following thermal decomposition is favoured (thermolysis):

(16) $${CO\left(NH_{2}\right)}_{2\left(s,l\right)}\to {NH}_{3\left(g\right)}+HNCO_{\left(g\right)}\;.$$

In this reaction, the urea decomposes into ammonia and isocyanic acid. Due to the high reactivity of HNCO, it was observed that its primary formation may subsequently lead to the formation of the compounds of higher molecular weight mentioned above [47]. In particular, the reaction with urea will lead to biuret, the reaction with itself (trimerization) will lead to cynuric acid, etc. (see [47]). To avoid the formation of these compounds, fast heating process is recommended to obtain only ammonia and isocyanic acid. As pointed out in [47] and elsewhere, this reaction is endothermic by +185.5 kJ/mol at standard conditions (298 K, 1 bar). The released gaseous ammonia can take part in the SCR reactions while the resulting isocyanic acid (HNCO) will produce ammonia, through hydrolyzation on the SCR catalyst (or in the gas phase at high temperatures) as follows:

(17) $${HNCO}_{\left(g\right)}+H_{2}O\to {NH}_{3\left(g\right)}+{CO}_{2\left(g\right)}\;.$$

While water evaporation and thermolysis processes are endothermic, the hydrolysis reaction is exothermic by −95.9 kJ/mol at standard conditions. As pointed out previously, a key issue faced by SCR systems is the inefficient performance resulting from incomplete thermolysis of urea ahead the SCR catalyst, among others. This incomplete thermolysis can be due to incomplete water evaporation or/and owning to thermolysis process itself which may lead to undesirable urea deposition on the walls and substrates inlets.

### 2.3. Large-Eddy Simulation

In the present numerical study, the solution domain consists of two parts, namely a fluid domain for flue gas transport and a 2-D thin film domain. In accordance to the procedure described in our previous works [31,48], the fluid part is governed by the balance equations for incompressible Newtonian fluid flow with variable physical properties and Fourier heat transport, while in the 2-D thin film domain incompressible multi-component liquid is transported with relevant sub-models for spray-wall impingement and film evaporation/decomposition. In LES context, the low-Mach number (Ma<0.3) formulation for the balance laws of mass, momentum and energy are employed for carrier phase and given as (see [49,50]):

(18) $$\frac{\partial \bar{\rho }}{\partial t}+\frac{\partial \bar{\rho }{\tilde{u}}_{i}}{\partial x_{i}}={\bar{S}}_{I,p}+{\bar{S}}_{I,f}$$

(19) $$\frac{\partial \bar{\rho }{\tilde{u}}_{i}}{\partial t}+\frac{\partial \bar{\rho }{\tilde{u}}_{i}{\tilde{u}}_{i}}{\partial x_{i}}=-\frac{\partial \bar{p}}{\partial x_{j}}+\frac{\partial {\bar{\sigma }}_{ij}}{\partial x_{j}}+\frac{\partial {\tilde{\tau }}_{ij}^{sgs}}{\partial x_{j}}+{\bar{S}}_{II,p}+{\bar{S}}_{II,f}$$

(20) $$\frac{\partial \bar{\rho }\tilde{h}}{\partial t}+\frac{\partial \bar{\rho }\tilde{h}{\tilde{u}}_{i}}{\partial x_{i}}=\frac{\partial }{\partial x_{j}}\left(\frac{\bar{\lambda }}{{\bar{c}}_{P}}\frac{\partial \tilde{h}}{\partial x_{j}}\right)-\frac{\partial {\tilde{q}}_{j}^{sgs}}{\partial x_{j}}+\sum_{k=1}^{N}\Delta h_{k}^{0}{\dot{\omega }}_{k}+{\bar{S}}_{III,p}+{\bar{S}}_{III,f}$$

(21) $$\frac{\partial \bar{\rho }{\tilde{\phi }}_{k}}{\partial t}+\frac{\partial \bar{\rho }{\tilde{\phi }}_{k}{\tilde{u}}_{i}}{\partial x_{i}}=-\frac{\partial {\tilde{J}}_{j}^{sgs}}{\partial x_{j}}+\bar{\rho {\dot{\omega }}_{k}}+{\bar{S}}_{I,p,k}+{\bar{S}}_{I,f,k}\;.$$

where ρ, $u_{i}$, *p*, *h* and $\phi_{k}$ are the density, velocity, pressure, enthalpy and scalar (i.e., species mass fraction) fields, respectively. The terms λ, $c_{P}$ and μ represent the thermal conductivity, the specific heat capacity and the viscosity, respectively. In case of reactive flow, the terms $h_{k}^{0}$ and $\omega_{k}$ are the enthalpy of formation and reaction rate. The source terms ($S_{I}$, $S_{II}$, $S_{III}$) due to spray droplet and film are represented by subscript *p* and *f* for corresponding governing equations of mass, momentum and energy.

In order to close the sub-grid stress term ${\tilde{\tau }}_{ij}^{sgs}$ in Equation (19), a one-equation model is used as proposed by Yoshizawa et al. [37]. It provides the necessary sub-grid kinetic energy $\left(k_{sgs}\right)$ to model when the droplet/particle-turbulence modulation arises. In Equation (21) the sub-grid scalar flux ${\tilde{J}}_{j}^{sgs}$ is modelled by a simple gradient approach by assuming the turbulent Prandtl and Schmidt number, $Pr_{t}=Sc_{t}=0.7$. The quantity $\bar{\rho {\dot{\omega }}_{k}}$ and $\sum_{k=1}^{N}\Delta h_{k}^{0}{\dot{\omega }}_{k}$ represent the net source terms due to the chemical reactions in species and heat equations. The highly non-linear chemical source term ${\dot{\omega }}_{k}$ is closed by a joint sub-grid scalar distribution obtained by means of the Eulerian stochastic field method [51]. To note is that ${\bar{S}}_{I}$, ${\bar{S}}_{II}$, ${\bar{S}}_{III}$ and ${\bar{S}}_{I,k}$ represent the volume-averaged source terms from dispersed fluid in mass, momentum energy and scalar equations, respectively.

### 2.4. Turbulence-Chemistry Interaction: Eulerian Stochastic Field (ESF)

As already pointed out in the previous section, the source terms due to chemical reactions ${\dot{\omega }}_{\alpha }$ may exhibit a high non-linearity. In addition to the resolved part, it is imperative to also consider the contributions of the turbulent fluctuations at the subgrid scale level along with the turbulence-chemistry interaction (TCI). In the LES context, such turbulence–chemistry interaction can be captured once a joint scalar sub-grid distribution is available at any given time [52]. To this purpose, the ESF method fulfils the requirement, as it is based on the transport equation of the Favre-filtered joint scalar probability density function $\tilde{P}$. The idea is to approximate $\tilde{P}$ as an ensemble of stochastic fields, which represent delta peaks in composition space. This requires the solution of a stochastic partial differential equation for each stochastic field $\zeta^{n}$ [41,51,53]

(22) $$\begin{array}{c} d\left(\bar{\rho }\zeta_{\alpha }^{n}\right)=\frac{\partial }{\partial x_{j}}\left(\bar{\rho }\zeta_{\alpha }^{n}u_{j}\right)dt+\frac{\partial }{\partial x_{i}}\left[\left(\frac{\bar{\mu }}{Sc}+\frac{\mu_{sgs}}{Sc_{sgs}}\right)\frac{\partial \zeta_{\alpha }^{n}}{\partial x_{i}}\right]dt\\ +\bar{\rho }{\dot{\omega }}_{\alpha }^{n}\left(\zeta^{n}\right)dt+{\bar{\dot{S}}}_{p,\alpha }+{\bar{\dot{S}}}_{f,\alpha }\\ +\frac{\bar{\rho }}{\tau_{t}}\left(\zeta_{\alpha }^{n}-{\tilde{\phi }}_{\alpha }\right)dt+\bar{\rho }\sqrt{\frac{2}{\bar{\rho }}\frac{\mu_{sgs}}{Sc_{sgs}}}\frac{\partial \zeta_{\alpha }^{n}}{\partial x_{j}}dW_{j}^{n}, \end{array}$$

In this equation *n* represents the number of stochastic fields to be solved for a scalar field α (i.e., species ($Y_{k}$) and enthalpy (*h*)), and $dW_{j}^{n}$ stands for the stochastic contribution to the equation in form of a Wiener process approximated by time-step increments $\eta_{i}^{n}\sqrt{dt}$, where $\eta_{i}^{n}$ is sampled from the dichotomic distribution {−1,+1}. The application of Eulerian stochastic field (ESF) to account for the turbulence–chemistry interaction in multi-phase reactive flow configuration such as SCR system is recently reported in a previous work [54], in which its importance for describing a reliable turbulence–chemistry interaction is highlighted in detail. Additionally, the complete solution procedure as adopted in OpenFOAM-v1612+ version is described by using a flowchart diagram [54]. All the simulations are carried out with a second order backward scheme to solve the transient term, while the second order Gauss linear scheme is utilized to solve both diffusion and convection term except the species convection part. The latter is solved by Gauss limitedLinear 1 scheme available in OpenFOAM.

### 2.5. Estimation of Entropy Production Rates

The entropy production rate derived from the filtered transport equation of entropy for multi-phase reactive flow system is formulated, according to [55] as:

(23) $$\frac{\partial \bar{\rho }\tilde{\eta }}{\partial t}+\frac{\partial }{\partial x_{i}}\left(\bar{\rho }\tilde{u_{i}}\tilde{\eta }\right)=\frac{\partial }{\partial x_{i}}\left(\bar{\rho }D_{m}\frac{\partial \tilde{\eta }}{\partial x_{i}}\right)-\frac{\partial }{\partial x_{i}}\left(\bar{\rho }\tau \left(u_{i},\eta \right)\right)+{\bar{\Pi }}_{v}+{\bar{\Pi }}_{q}+{\bar{\Pi }}_{d}+{\bar{\Pi }}_{ch}+{\bar{\Pi }}_{P}\;,$$

where the first terms on the left-hand side represent the local entropy change, entropy convection and the flux of entropy density η, respectively. The last five terms on the right-hand side express the filtered entropy production rate by viscous dissipation $\Pi_{v}$, heat dissipation $\Pi_{v}$, mass diffusion $\Pi_{d}$, chemical reaction $\Pi_{ch}$ and contribution due to the presence of dispersed phase $\Pi_{P}$. According to [20,32,50], and within the LES context, the temporal averaged filtered entropy production rates can be calculated as the sum of the resolved and residual part for $\Pi_{v}$, $\Pi_{H}$, and $\Pi_{d}$ as:

(24) $${\bar{\Pi }}_{v}=\bar{\frac{1}{T}\tau_{ij}\frac{\partial u_{i}}{\partial x_{j}}}=\underset{{\bar{\Pi }}_{v}^{res}}{\underset{︸}{\frac{\bar{\mu }}{\bar{T}}\left(\frac{\partial {\bar{U}}_{i}}{\partial x_{j}}+\frac{\partial {\bar{U}}_{j}}{\partial x_{i}}\right)\frac{\partial \bar{U_{i}}}{\partial x_{j}}}}+\underset{{\bar{\Pi }}_{v}^{sgs}}{\underset{︸}{\frac{\bar{\rho }}{\bar{T}}\frac{\nu_{sgs}^{3}}{\Delta^{4}C_{S}^{4}}}}$$

(25) $${\bar{\Pi }}_{q}=\bar{\frac{\lambda }{T^{2}}\frac{\partial T}{\partial x_{i}}\frac{\partial T}{\partial x_{i}}}=\underset{{\bar{\Pi }}_{q}^{res}}{\underset{︸}{\frac{\bar{\lambda }}{{\bar{T}}^{2}}\frac{\partial \bar{T}}{\partial x_{i}}\frac{\partial \bar{T}}{\partial x_{i}}}}+\underset{{\bar{\Pi }}_{q}^{sgs}}{\underset{︸}{\frac{4{\bar{\rho c}}_{p}\nu_{sgs}}{3C_{OC}\pi^{4/3}C_{s}^{4/3}Pr{\bar{T}}^{2}}\frac{\partial \bar{T}}{\partial x_{i}}\frac{\partial \bar{T}}{\partial x_{i}}}},$$

(26) $${\bar{\Pi }}_{d}=\bar{\frac{\lambda }{c_{p}}\sum_{k=1}^{Ns}\frac{R_{k}}{Y_{k}}\frac{\partial Y_{k}}{\partial x_{i}}\frac{\partial Y_{k}}{\partial x_{i}}}=\underset{{\bar{\Pi }}_{d}^{res}}{\underset{︸}{\frac{\bar{\mu }}{Sc}\sum_{k=1}^{Ns}\frac{R_{k}}{{\bar{Y}}_{k}}\frac{\partial {\bar{Y}}_{k}}{\partial x_{i}}\frac{\partial {\bar{Y}}_{k}}{\partial x_{i}}}}+\underset{{\bar{\Pi }}_{d}^{sgs}}{\underset{︸}{\sum_{k=1}^{Ns}\frac{R_{k}\bar{\rho }}{{\bar{Y}}_{k}}\frac{2}{3C_{OC}\pi^{4/3}C_{s}^{4/3}}\frac{\nu_{sgs}}{Sc}\frac{\partial {\bar{Y}}_{k}}{\partial x_{i}}\frac{\partial {\bar{Y}}_{k}}{\partial x_{i}}}},$$

with $C_{OC}=1.34$ expressing the Obukhov–Corrsin constant [56], $C_{S}$ the Smagorinsky coefficient [57] and Δ the filtered width.

The application of the Eulerian Stochastic field method for the description of the turbulence–chemistry interaction allows a direct closure of the reaction source term. Thus, the formulation of the total entropy production due to the chemical reaction can be written as:

(27) $${\bar{\Pi }}_{ch}=-\frac{1}{T}\sum_{k=1}^{N}\mu_{k}{\dot{\omega }}_{k}\;.$$

The presence of dispersed phase requires the relevant exchange of species, mass, momentum and heat between the phases in presence. Accordingly, the entropy generation rate due to the spray dynamics in the carrier phase is formulated as follows [58,59]:

(28) $$\Pi_{P}=g_{III}+g_{III}+g_{I,kin}+g_{I,chpot}$$

(29) $$g_{III}=\frac{1}{T}S_{III},g_{II}=-\frac{1}{T}u_{i}S_{II,i},g_{I,kin}=\frac{1}{T}\frac{u_{i}u_{i}S_{I}}{2},g_{I,chpot}=-\frac{1}{T}\mu_{V}S_{I}\;.$$

In these equations, the terms $g_{I,kin}$, $g_{II}$, $g_{III}$ and $g_{I,chpot}$ represent the entropy production due to the evaporated mass kinetic energy, the momentum exchange between spray and carrier phase, the spray evaporation, and the evaporated mass chemical potential, respectively. To preserve the brevity of this paper, the details of the 2-D thin film approach formulation for capturing the film dynamics are not described here, more details can be found in a previous contribution [60].

## 3. Numerical Configuration: A Generic SCR System

**Figure 1:**
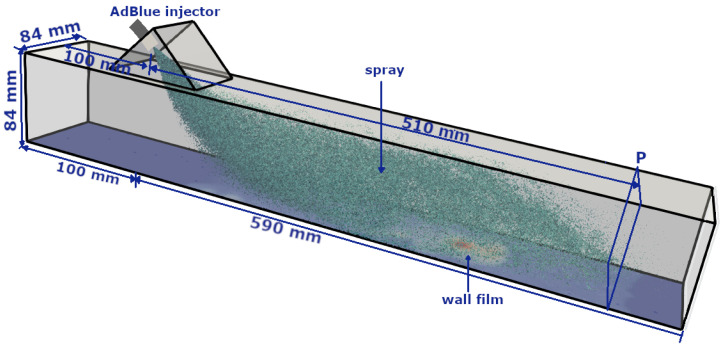
The generics SCR configuration [60]; numerical domain with dimensions and AdBlue injector (45${}^{\circ }$ injection direction in horizontal axis).

In this work, a generic SCR configuration representing a test bench featuring a simple geometric configuration of cross-section of 84 mm × 84 mm is chosen (see Figure 1). It is especially designed to support the numerical model development and validation for processes relevant in a SCR system under varied operating conditions [60,61,62]. It is expected that, this experimental setup will allow the measurements related to the flow characteristics, the species concentration, the AdBlue film thickness, the deposit formation etc. Table 1 provides the selected operating parameters in this study, in which the influence of gas flow rate and temperature are especially analysed. The cases “CW1” and “CW2” represent the operating conditions with water injection [62]. For these both cases, the measurement data of the “H2O” species concentration and the temperature profile, on the cross-section at 510 mm (see plane “P” in Figure 1) downstream the injection location, are available. The cases “C1–C4” represents the more realistic SCR operating conditions with AdBlue injection [60]. The water/AdBlue is injected at constant injection rate of 11.7 kg/h with spray angle of 45${}^{\circ }$, while the injection duration $t_{d}$ is varied based on the carrier gas mass flow rate. The test bench also features an extended upstream length of 25D (D = 84 mm) to provide a fully developed turbulent flow to the inlet of measurement region (see Figure 2 in [60]). Therefore, instead of considering the extended upstream length, a digital filtered inlet method proposed by Klein et al. [63] is used to insure a fully turbulent boundary condition at the inlet in computational domain shown in Figure 1. This allows us to consider the smaller computational domain and thereby considerably reduce the total computational cost as pointed out in previous study [54]. A fully conformal hexahedral mesh with approx. 2.35 millions control volumes (CVs) is utilized for the spatial discretization of the primary carrier phase domain, while 102,000 CVs are utilized for the 2-D film domain. The reliability of the chosen mesh resolution has already been verified based on various LES quality of index criteria in [54] for the SCR configuration and operating conditions also relevant to this work. The liquid injection and the relevant spray dynamics in each injection event are solved by using the computational parcel injection of 10,000,000/s. In this study, interaction between gas and liquid film is accounted by mapped momentum, heat and mass flux boundary conditions along the gas–film interface. A varying temperature boundary condition is applied at the film–wall interface based on the measurement in [60]. More details about this generic SCR configuration and measurement techniques can found in [60,61,62].

## 4. Results and Discussion

Even though the SCR system has been primarily employed for diesel NOx reduction, there is no comprehensive data set available to validate the numerical models for all relevant physical phenomena (such as turbulent exhaust flow, AdBlue injection/atomization, spray–wall interaction, film formation, multi-phase reaction, NOx reduction etc.) in a realistic SCR configuration. Therefore, the respective models validation and verification are carried out in an individual test bench. The integrated numerical approach is then utilized to carry out more detailed numerical investigation in more realistic SCR configuration [8]. However, in this study the individual numerical modules (e.g., AdBlue injection, spray–wall–interaction/film formation, wall–film/spray evaporation, species transport, reactions etc.) are being developed and then validated by comparison with the measurement data from the same SCR configuration [60].

In this study, the model validation is carried out for multi-phase fluid flow phenomena including the AdBlue and water injection in a generic SCR configuration (see Figure 1, [60,62]) for only the selected operating conditions. Subsequently, the numerical analysis is carried out for more realistic SCR operating conditions as reported in [60]) that also includes AdBlue injection and resulting urea decomposition with NH_3_ conversion. Finally, the estimation of the entropy production of the individual processes is carried out using the respective formulation as provided in Equations (24)–(29).

### 4.1. Model Validation

**Figure 2:**
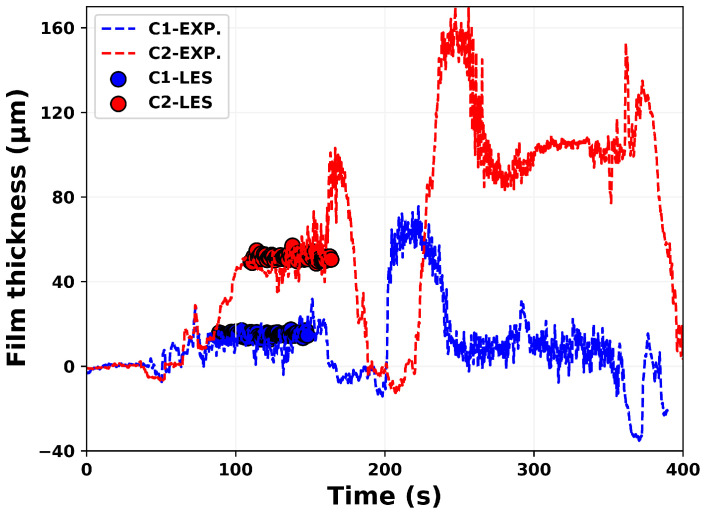
Validation of film evolution for the cases C1 and C2 with experiments [60].

**Figure 3:**
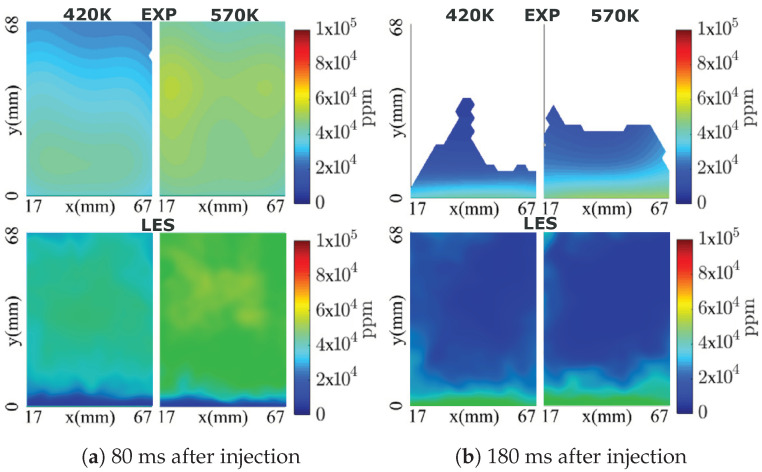
Comparison of H_2_O species concentration profile at plane “510 mm” downstream to the injector for two operating conditions ($U_{b}=3$ m/s, $T_{g}=420$ K and $U_{b}=3$ m/s, $T_{g}=570$ K) [62].

**Figure 4:**
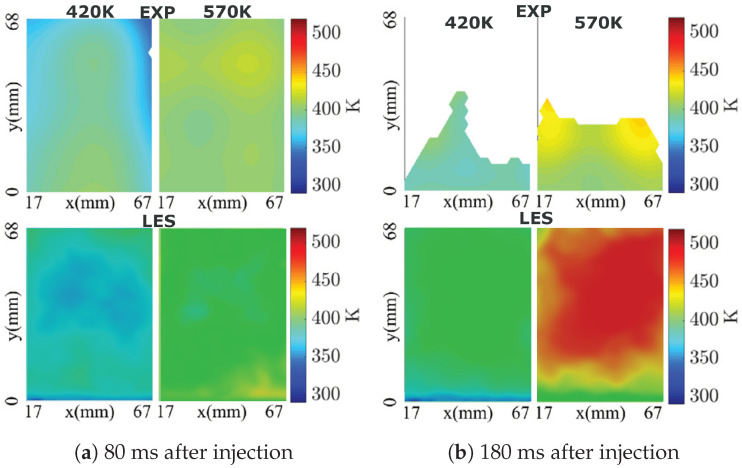
Comparison of temperature profile at plane “510 mm” downstream to the injector for two operating conditions ($U_{b}=3$ m/s, $T_{g}=420$ K and $U_{b}=3$ m/s, $T_{g}=570$ K) [62].

In order to validate the adopted numerical methodology, first the measurement data for the evolution of AdBlue film thickness on the bottom duct wall over many injection events as reported in [60] is used. The processes of spray–wall interaction and film dynamics under high temperature conditions essentially require a conjugate heat transfer approach to take properly in to account the thermal inertia of duct material. This is not considered in this study due to associated high computational cost. In fact simulations are carried out with pre-initialized film thickness and wall temperature suggested by experimental measurement, and thus representing a scenario of wet-wall splashing. Subsequently simulations are carried out for total 60 and 54 injection events for cases C1 and C2, respectively. Figure 2 depicts the AdBlue film thickness plotted for many injection cycles for the two operating conditions C1 and C2. The respective film thickness profiles obtained with LES are shown in solid circles. The compared results show a reasonable agreement for the wall film thickness, especially for the scenario of developed AdBlue wall film. A detailed numerical validation has already been carried out for transient evolution of film thickness for both early stage and late injection events for one operating condition ($T_{g}$ = 180 °C and $V_{g}$ = 6.5 m/s) in [54,60]. It should be noted that, the film evolution features highly transient and complex phenomena that essentially depend on the surface properties, operating conditions and thermo-chemical state of the film mass (see also Figure 2). In addition, localized point measurements are carried out for film build-up during multi-cycle AdBlue injection (1 Hz injection frequency). This leads to the difficulties associated with the reproducibility by experimental measurements as reported in [60]. Second, the available measurement data (cases “CW1” and “CW2”, in which only water is injected) for H_2_O species mass fraction and temperature profile are employed for a further evaluation of the numerical methodology [62]. The phased averaged data were obtained for 10 consecutive injection events with injection frequency of 1 Hz. Water was injected with a constant injection rate of 11.7 kg/h for 40 ms of injection duration and hence the mean injection rate of ≈500 g/h. In line with the experiment, the numerical simulations are also carried out for 10 consecutive injection cycles and the LES results are obtained by phase averaging the H_2_O species mass fraction and temperature profile in the cases “CW1” and “CW2” for two instances (80 ms and 180 ms after injection) on the plane at 510 mm downstream the injector nozzle (see plane “P” in Figure 1). The comparison of the LES results with the experimental data for the H_2_O species mass fraction is shown in Figure 3. A strong impact of the gas phase temperature can be clearly seen in overall species distribution along the duct section with obviously higher mass fraction of H_2_O for higher carrier gas temperature due to the enhanced evaporation. Additionally, the distribution of H_2_O mass fraction looks more uniform at time 80 ms after injection. This can be attributed to the convection of evaporated H_2_O which is further mixed during the spray evolution. Subsequently, at instance 180 ms after injection, the higher mass fraction is observed primarily along the SCR duct wall indicating the evaporated H_2_O mass has already convected beyond the plane of interest (z = 510 mm) and the visible H_2_O vapour is supplied by evaporation of liquid film and/or by the evaporation of spray droplets slowed down along the wall due to the spray–wall interaction and the boundary layer flow. These trends are clearly visible in both experiment and LES obtained results suggesting a reasonable agreement especially by also considering the observed range of H_2_O mass fraction in LES and experiment. However, there is a visible deviation near the bottom duct wall for instance of 80 ms after injection as almost no H_2_O vapour is present in this region. It can be partly attributed to the complex nature of the wall film dynamics as highlighted earlier and partly to the delayed transport of water vapour, similar to the later instance of 180 ms after injection. Similar observation can be also made for the corresponding temperature profiles as shown in Figure 4. A lower phase temperature is observed for instance of 80 ms after injection suggesting the utilization of gas phase sensible heat to evaporate the water droplets. Subsequently, the gas phase temperature is low along the SCR duct wall that corresponds to film and/or droplet evaporation along the wall. However, apart from the temperature over-prediction in LES, there is obvious difficulty for direct and quantitative comparison of both LES and the experimentally obtained temperature profile especially at later stage due to the unavailability of complete measurement data. It should be also observed here that the comparison is performed only for 10 injection events which remains too small to carry out a reliable comparison when considering the highly transient and complex nature of the flow dynamics.

### 4.2. AdBlue Injection and SCR Flow Dynamics

**Figure 5:**
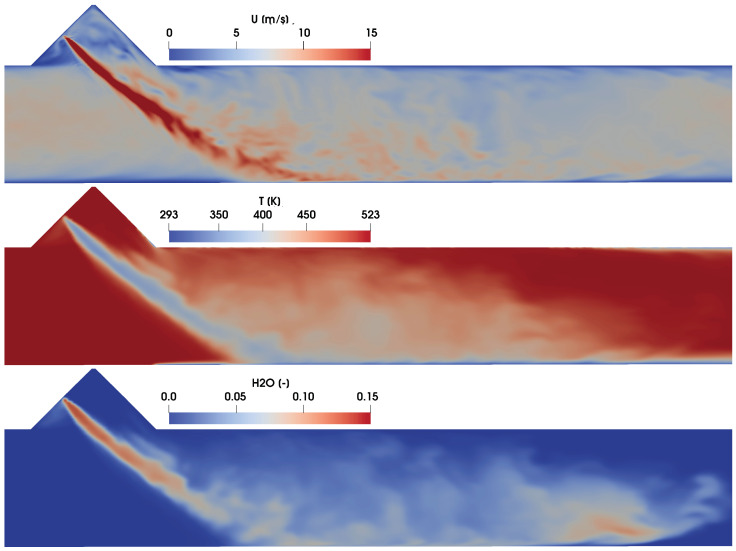
Instantaneous velocity, temperature and H_2_O mass fraction profile for case C2 at SOI = 50 ms.

Further simulations are conducted with more realistic operating conditions similar to the engine SCR operations as listed in Table 1 (C1–C4, [60]). These will assist us to carry out the entropy generation analysis by evaluating the impact of the operating conditions, namely the cross flow velocity and the exhaust gas temperature. In this regards, Figure 5 represents the instantaneous velocity, the temperature and H_2_O mass fraction profiles at time SOI = 50 ms (SOI is the time from the start of the injection) for case “C2” along the middle sectional plane. A strong impact of AdBlue injection can be observed in the velocity distribution where the spray injection imparts momentum on the carrier phase due to the drag force resulting in a higher gas velocity with a visible high velocity gradient along the spray surfaces. In combination with spray atomization, the cross flow further enhances the droplet evaporation by means of the sensible heat while cooling the exhaust gas as seen in the obtained temperature profile (see Figure 5, middle). The corresponding H_2_O vapour profile depicted in Figure 5 (bottom), shows that during the initial stage of evaporation, the water is mostly evaporated from the AdBlue droplets due to the relatively higher volatility compared to the urea. It should be also noted that these thermo-fluid flow features determine the respective contribution towards the total entropy production under these specific operating conditions as discussed in the following sections.

### 4.3. Predictions of Entropy Production Rates

**Figure 6:**
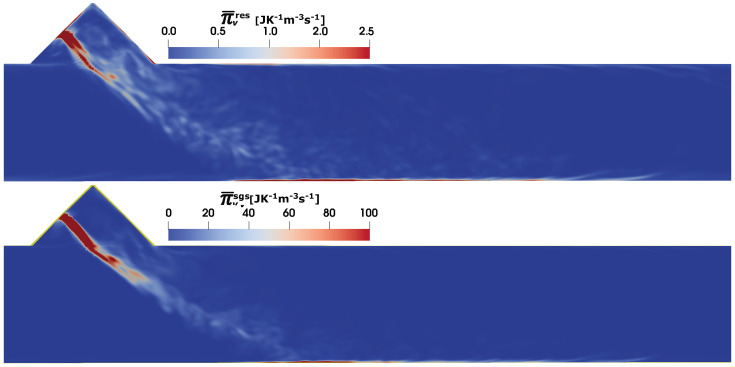
Entropy generation rate due to viscous dissipation ${\bar{\Pi }}_{v}$ in SCR duct (C2) at SOI = 50 ms; resolved (**top**), sub-grid scale (**bottom**).

To quantify the different entropy generation source terms, the Equations (24)–(29) are used. It is worth mentioning that, the entropy generation analysis due to the film dynamics, within the film/droplet mass are not considered and rather left for a future study. The evaluation of the generated entropy will be then analysed only in the gas phase. In this regard, Figure 6 shows the estimated profile of the entropy production rate due to the viscous dissipation (see Equation (24)) at SOI = 50 ms along the middle sectional plane. As pointed out in some previous contributions in the research group of authors [21,33,34,49], the entropy generation is essentially a sub-grid scale phenomena and the total entropy production associated with sub-grid scale is considerably higher. As evident in Figure 6, a higher value can be seen along the spray surface and spray impingement region characterized by a strong velocity gradient.

**Figure 7:**
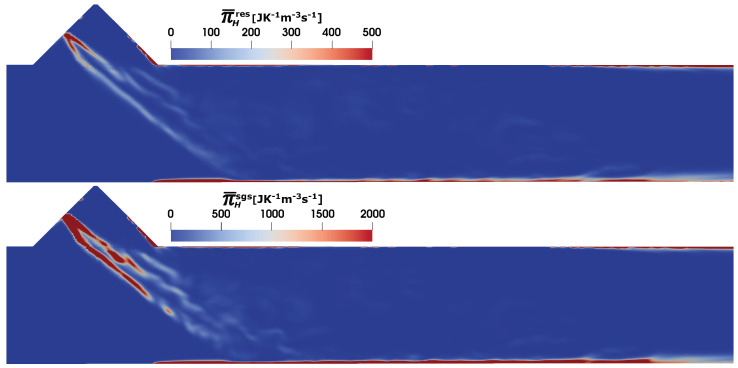
Entropy generation rate due to heat dissipation ${\bar{\Pi }}_{H}$ in SCR duct (C2) at SOI = 50 ms; resolved (**top**), sub-grid scale (**bottom**).

Next, the entropy generation rates due to heat dissipation (see Equation (25)) are shown in Figure 7. Similar to the viscous dissipation, the sgs contribution to the entropy generation dominates the total heat entropy generation source term. The higher temperature gradients along the spray surface and the duct wall (see also the temperature profile in Figure 5) are mainly responsible for the higher entropy production due to heat dissipation. It should be noted that the presence of the wall film and its evaporation induces a large temperature gradient near the SCR duct wall.

**Figure 8:**
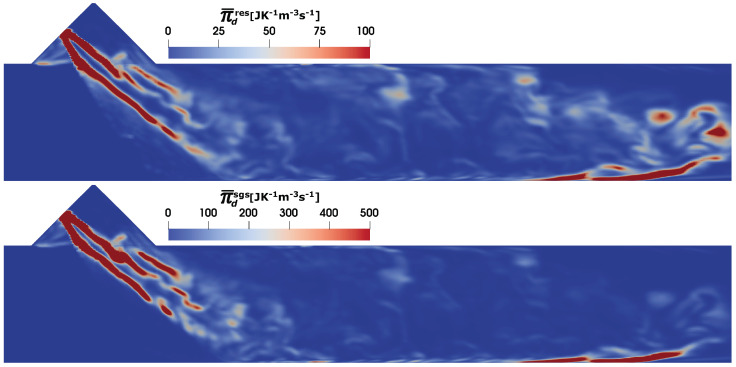
Entropy generation rate due to species mixing ${\bar{\Pi }}_{d}$ in SCR duct (C2) at SOI = 50 ms; resolved (**top**), sub-grid scale (**bottom**).

Similar to many energy or reactive system, mixing or diffusion of species mass play a vital role also in SCR DeNOx system. In ideal situation, the reactive flow system should be uniformly/fully mixed with minimal exergy loss or minimal entropy production. The proper mixing is accomplished either by additional mixing element or flow induced mixing. Figure 8 depicts the entropy production due to the mixing process (see Equation (26)). The profile of the entropy generation rate is closely linked to the profile of large species gradient induced during the evaporation of AdBlue droplets (see Figure 5, bottom). As it is the case with viscous and heat dissipation, a large part of entropy generation due the mixing is produced at the sub-grid scale.

**Figure 9:**
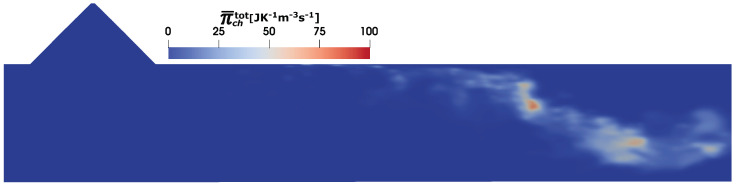
Entropy generation rate (true value: combined resolved and sub-grid scale value) due to chemical reaction ${\bar{\Pi }}_{ch}^{tot}$ in SCR duct (C2) at SOI = 50 ms.

In this study, the urea decomposition and NH_3_ conversion are achieved by two-step global mechanism, while the ESF based approach provides the fully closed reaction rate terms, which facilitates the estimation of the total (sgs+resolved) entropy production due to the chemical reaction (see Equation (27)) as shown in Figure 9. Since the urea decomposition can largely be initiated once the water is fully evaporated from the droplets, the associated two-step reaction kinetics are often delayed. This is also visible with the obtained result of entropy production due to chemical reaction, which resembles mostly the NH_3_ conversion profile.

**Figure 10:**
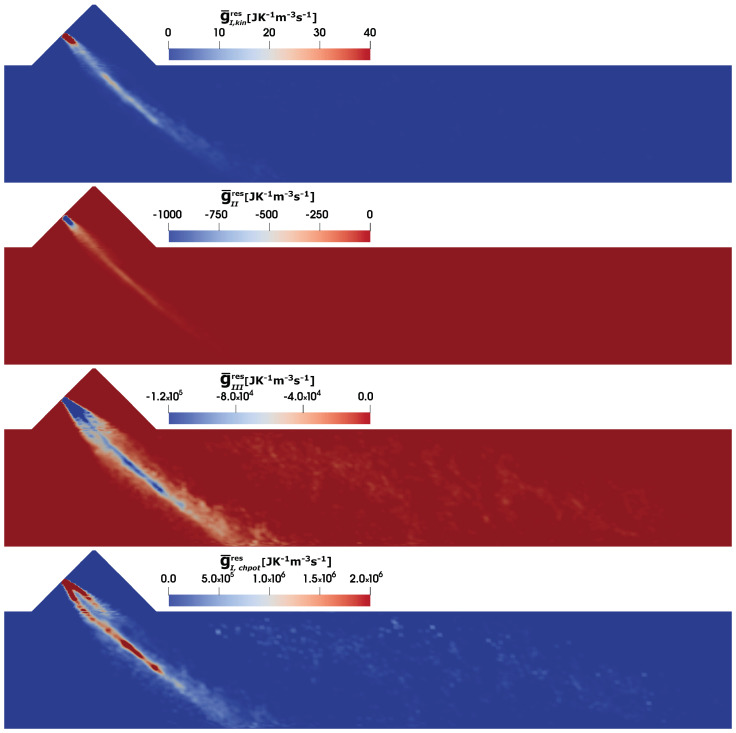
Contributions to the entropy generation rate because of AdBlue injection in SCR duct (C2) at SOI = 50 ms due to the evaporated mass kinetic energy ${\bar{g}}_{I,kin}$, the momentum exchange between spray and carrier phase ${\bar{g}}_{II}$, the spray evaporation ${\bar{g}}_{III}$ and the evaporated mass chemical potential ${\bar{g}}_{I,chpot}$ (from **top** to **bottom**).

Finally, the evaluation of the entropy production (see Equation (28) and (29)) due to the presence of AdBlue spray dynamics is carried out. Figure 10 depicts the instantaneous profile of individual contributions due to the spray dynamics for case C2 at at SOI = 50 ms. In particular, the entropy generation rate associated with the evaporated mass kinetic energy, the momentum exchange between the spray and the carrier phase, the spray evaporation and the evaporated mass chemical potential, respectively, (from top to bottom). The mass addition in the carrier phase due to the evaporation of the spray droplets is relatively small and hence the associated entropy production contribution ${\bar{g}}_{I,kin}$ is considerably small but positive. However, the momentum exchange between the spray droplets and the carrier phase results in a negative entropy due to the slip velocity in particular close to the injection region. In addition, no entropy is produced in the downstream region since the slip velocity between the dispersed phases becomes smaller/zero. For the spray evaporation process, the considerable quantity of sensible heat taken from the exhaust gas and used to evaporate the spray droplets, results in a relatively higher entropy generation rate but negative (or entropy destruction). Compared to the evaporated mass kinetic energy, the momentum exchange between the spray and the carrier phase and the spray evaporation entropy source terms, the chemical potential of evaporated mass contribution exhibits the higher values of entropy generation rates.

**Figure 11:**
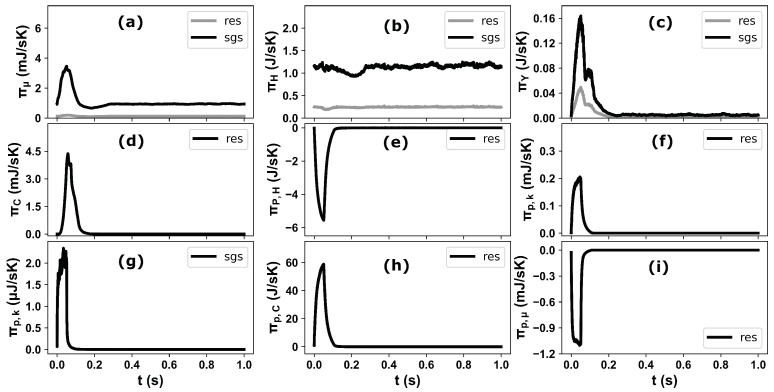
The total entropy generation for individual processes in a the SCR domain during one injection event for Case-2: (**a**) viscous dissipation, (**b**) heat dissipation, (**c**) mixing, (**d**) chemical reaction, (**e**) droplet evaporation, (**f**) due evaporated mass kinetic energy (resolved), (**g**) due evaporated mass kinetic energy (sub-grid), (**h**) evaporated mass chemical potential and (**i**) due to momentum exchange between droplet and carrier phase; res (resolved), sgs (sub-grid scale).

**Figure 12:**
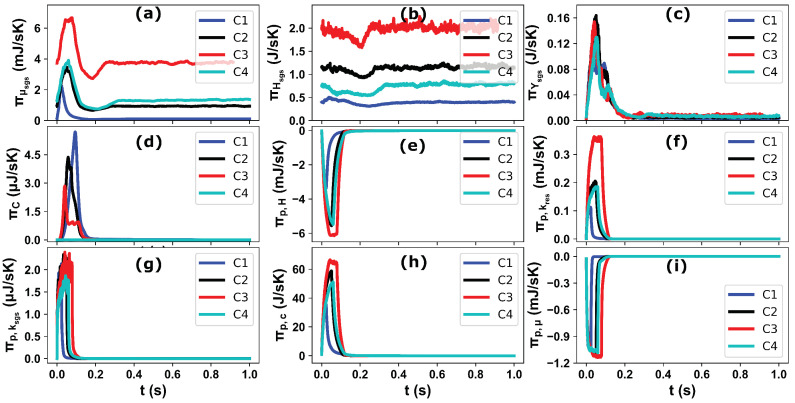
Comparison of the total entropy generation for individual processes in a the SCR domain during one injection event for all 4 operating conditions: (**a**) viscous dissipation (sgs only), (**b**) heat dissipation (sgs only), (**c**) mixing (sgs only), (**d**) chemical reaction, (**e**) droplet evaporation, (**f**) evaporated mass kinetic energy (resolved), (**g**) evaporated mass kinetic energy (sub-grid), (**h**) evaporated mass chemical potential and (**i**) momentum exchange between droplet and carrier phase.

The operating conditions in the selected test cases vary in terms of the flue gas velocity with temperature, injected AdBlue mass and injection duration. Therefore, one to one comparison and analysis of the local entropy generation rate may not provide a transient and global perspective about these processes, especially to optimize the complete process/design parameters based on the entropy generation minimization (EGM) approach. To this purpose, the contributions of the individual processes on entropy generation are firstly evaluated for one injection event for case “C2” (see Figure 11). It should be noted that, here the total entropy generation/destruction represents the integration over whole domain at each simulation time step. The obtained results of the resolved (res) and sub-grid-scale (sgs) parts of entropy generation certify that the most of entropy productions are associated with the processes at sub-grid-scale level. This hold especially true for species mixing, viscous and heat dissipation. The visible peak can be also observed for the entropy production/destruction during the AdBlue injection and the spray/species evolution within the whole length of the computational domain. As expected, the entropy production due to the chemical potential of evaporated species during the phase change process is significantly higher than the other processes, while the entropy destruction is observed for both heat and momentum exchanges during the AdBlue spray dynamics with considerably higher rates attributed to the heat exchange due to evaporation. As mentioned above, to maintain the brevity of this work and to limit the irreversibility analysis within the carrier phase, the respective entropy destruction/production within the spray droplets and AdBlue film domain are not considered in the present analysis. This aspect is devoted to a future work. Subsequently, the impact of operating parameters (flue gas velocity, temperature and AdBlue injection) on the entropy generation associated with the involved processes are then analysed (see Figure 12) for the selected cases. In particular, the influence of flue gas temperature can be readily observed among the cases C2 and C4, where the entropy production due to the chemical reaction is negligible in C4 due to the lower carrier phase temperature (*T_g_* = 180 °C), while it is higher for the case with lower carrier gas velocity (C1) due to the increased residence time for the reactions to proceed. The entropy generation due to the viscous dissipation and momentum exchange with dispersed phase are of similar order for the cases C2 and C4 due to the identical bulk flow velocity and similar pattern of AdBlue injection. The case C3 involves a higher flue gas velocity (*V_g_* = 10 m/s) and temperature (*T_g_* = 250 °C) which have a strong impact on the entropy productions as revealed in Figure 12.

## 5. Conclusions

In this study, a detailed entropy generation analysis based numerical investigation was carried out to analyse the contributions of the various processes involved in a SCR-DeNOx System. In particular, the impact of heat transfer, fluid flow, phase change, mixing and chemical reaction due to AdBlue injection on the entropy generation of the carrier phase is investigated for the first time in a SCR DeNOx system. The adopted numerical modules are validated by comparison with the available measurement data for AdBlue and water injection cases. Subsequently, the validated numerical approach is used to carry out a numerical analysis of the selected operation points ($T_{g}$, $V_{g}$) in a generic selective catalytic reduction (SCR) configuration. From this study, following inferences can be drawn:

- The entropy production is essentially a sub-grid-scale process as proven by the results of the viscous dissipation, heat dissipation and mass diffusion contributions.
- The impact of the spray dynamics is significant near the injection region through the modified thermal flow field and their gradient.
- Apart from the modified thermal profile in injection region, the higher entropy production due to heat dissipation is also associated with the near wall phenomena such as wall–film dynamics and spray transport close to wall region after spray–wall interaction.
- The total entropy production due to the chemical reaction is relatively low caused by the incomplete urea decomposition and NH_3_ conversion.
- The entropy contribution associated with the kinetic energy of evaporated mass features the lowest values because of the overall reduced evaporated mass. However, the negative entropy production rates (entropy destruction) are observed due to the slip velocity between the dispersed phase $\bar{g_{II}}$ and the utilization of the gas phase sensible heat during the spray droplet evaporation $\bar{g_{III}}$. The entropy production due to the chemical potential of evaporated mass is the most significant compared to other processes related to the spray dynamics.
- Depending upon the respective AdBlue injection duration and injected mass the effects of the operating parameters ($T_{g}$, $V_{g}$) on the entropy production is closely linked through the evolved flow, thermal, phase change, mixing and chemical reaction processes.

## Figures and Tables

**Table 1:** The operating parameters for numerical investigation [60,62].

Case	$T_{g}$ (°C)	$V_{g}$ (m/s)	${\dot{m}}_{g}$ (kg/h)	${\dot{m}}_{\mathrm{DEF}}$ (g/h)	*t*${}_{d}$ (ms)	*Re* ${}_{g}$
CW1	147	3.0	64	500	≈40	8950
CW2	297	3.0	47	500	≈40	2817
C1	250	3.0	51	278	≈24	6800
C2	250	6.5	111	604	≈51	14,800
C3	250	10	171	929	≈79	22,300
C4	180	6.5	129	697	≈60	17,600

## Abbreviations

The following abbreviations are used in this manuscript:

EGAS	exhaust-gas-after-treatment system
BEV	Battery Electric Vehicle
EGM	Entropy Generation Minimization
LES	Large-Eddy Simulation
RANS	Reynolds-averaged Navier–Stokes
SCR	Selective Catalytic Reduction
CFD	Computation Fluid Dynamics
ESF	Eulerian Stochastic Field
TCI	Turbulence–chemistry interaction
